# Acousto-Optic–Based Wavelength-Comb-Swept Laser for Extended Displacement Measurements

**DOI:** 10.3390/s17040740

**Published:** 2017-03-31

**Authors:** Nam Su Park, Soo Kyung Chun, Ga-Hee Han, Chang-Seok Kim

**Affiliations:** 1 Department of Advanced Circuit Interconnection, Pusan National University, Busan 46241, Korea; ns.park@pusan.ac.kr; 2 Department of Cogno-Mechanics Engineering, Pusan National University, Busan 46241, Korea; sootnrud@pusan.ac.kr (S.K.C.); gahee@pusan.ac.kr (G.-H.H.); 3 Advanced Circuit Interconnection Division, Samsung Electro-Mechanics, Busan 46754, Korea; namsu89.park@samsung.com

**Keywords:** laser and laser optics, swept laser, acousto-optic tunable filter, Fabry-Pérot etalon filter, point spread function, interferometric displacement measurement

## Abstract

We demonstrate a novel wavelength-comb-swept laser based on two intra-cavity filters: an acousto-optic tunable filter (AOTF) and a Fabry-Pérot etalon filter. The AOTF is used for the tunable selection of the output wavelength with time and the etalon filter for the narrowing of the spectral linewidth to extend the coherence length. Compared to the conventional wavelength-swept laser, the acousto-optic–based wavelength-comb-swept laser (WCSL) can extend the measureable range of displacement measurements by decreasing the sensitivity roll-off of the point spread function. Because the AOTF contains no mechanical moving parts to select the output wavelength acousto-optically, the WCSL source has a high wavenumber (*k*) linearity of *R*^2^ = 0.9999 to ensure equally spaced wavelength combs in the wavenumber domain.

## 1. Introduction

Wavelength-swept laser (WSL) sources have been developed to be a powerful tool for the application of optical sensing and imaging techniques, such as optical Fourier-domain reflectometry (OFDR) and swept-source optical coherence tomography (SS-OCT) [1,2,3]. The performance of the wavelength-swept laser can be determined by various factors, such as the spectral bandwidth, linewidth (or coherence length), swept repetition rate, center wavelength and linearity in wavenumber (*k*). Many studies have been performed to achieve better wavelength-swept laser performance with a broad spectral bandwidth, narrow linewidth, high swept repetition rate, various center wavelengths, and *k*-linear sweeping. 

Among the various factors affecting wavelength-swept lasers, the narrowing of the spectral linewidth is especially important for increasing the interferometric displacement measurement range in OFDR and SS-OCT by improving the coherence length of the light source [3]. Recently, optical-domain sub-sampled OCT has been proposed to measure larger depth ranges and reduce the data acquisition bandwidth simultaneously [4,5,6,7,8,9,10]. There has been a limitation in reducing the linewidth in the conventional continuous-spectrum broadband light source of spectral-domain optical coherence tomography (SD-OCT) and the continuous wavelength-swept laser source of SS-OCT. Therefore, a spectrally sampled broadband light source for SD-OCT and a wavelength-comb-swept laser (WCSL) source for SS-OCT can be used to reduce optical interference fringe decay and extend the interferometric displacement measurement range because of their discretely distributed comb-like spectral information with fixed *k*-space periodicity [4,5,6,7,8,9,10].

Most WCSLs use two intra-cavity filters, a tunable wavelength-selection filter (TWSF) and a Fabry-Pérot etalon filter [5,6,7,8,9,10]. The TWSF is employed for the tunable selection of the output wavelength along repeated time and the etalon filter for the narrowing of the spectral linewidth at each wavelength with fixed *k*-space periodicity. In general, the wavelength sweeping of WCSL can be simply achieved by mechanical moving components, such as a fiber Fabry-Pérot tunable filter (FFP-TF) [5,6,7] and a polygon mirror scanner [8]. These mechanically sweeping parts are known to suffer from the disadvantages of non-linear tuning of the wavenumber and environmental instability. If the WCSL does not meet the *k*-linearity requirement, it is necessary to resample and recalibrate the non-linear wavenumber spacing of the interferometer spectrum [11,12,13].

In this research, the higher *k*-linearity of the WCSL output is demonstrated based on an acousto-optic tunable filter (AOTF) because the AOTF is not related with mechanical movement but operated by electric signals into the acoustic crystal. The output wavelength is inversely proportional to the frequency of the applied radio frequency (RF) signal in the AOTF. A comb-like spectral laser source with a narrow spectral linewidth is implemented for extended interferometric displacement measurements by combining an AOTF [14] and an etalon filter [15,16,17,18]. 

## 2. Generation of Comb-Like Spectral Laser Source with Narrow Spectral Linewidth

Owing to the dense wavelength spacing of the multiple longitudinal modes in a long laser cavity, the broad linewidth of the laser peak in the conventional WSL corresponds to a limited displacement measurement of less than 6 mm of the point spread function (PSF) by monitoring the decreased sensitivity roll-off [14]. When we insert an etalon filter with a periodic bandpass spectrum into the fiber cavity to partially suppress the multiple longitudinal modes in the cavity, the narrowing of the spectral linewidth can be easily implemented to extend the coherence length of the laser light. However, the comb-like spectrum is generated when the free spectral range (*FSR*) of the etalon filter is smaller than the linewidth of the laser peak in static operation. If the center wavelength of the laser peak is swept with the shifting wavelength spectrum of the AOTF, the output power will be repeatedly generated according to the *FSR* of the etalon filter in the time domain.

The *FSR* is the spacing of the transmission peaks due to the cavity length, which can be a fiber cavity for the longitudinal mode (*FSR_LM_*) or the distance between two plane-parallel surfaces for an etalon filter (*FSR_EF_*). The value of *FSR* is simply defined as [15,16];

(1) $$FSR=\frac{c}{2nL}$$

where *c* is the speed of light, *n* is the refractive index of the medium, and *L* is the cavity length. The etalon filter is used as a comb filter based on the Fabry-Pérot interferometer. It consists of two plane-parallel surfaces that are positioned at a fixed distance (*L*) from each other and is equipped with a partially reflective coating. 

**Figure 1:**
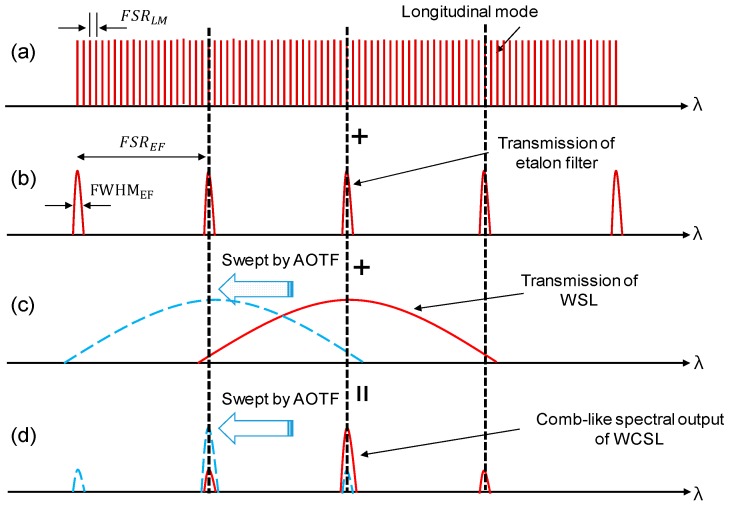
Generation of comb-like spectral laser source with narrow spectral linewidth by combining an etalon filter and an AOTF. (**a**) Longitudinal mode position corresponding to the round trip of the laser cavity; (**b**) transmission of the etalon filter; (**c**) transmission of conventional WSL; (**d**) comb-like spectral output of the WCSL. The transmission spectra swept by the AOTF for both the WSL and WCSL are also shown with dashed lines in (**c**,**d**), respectively.

Figure 1 shows a schematic diagram of the generation of the comb-like spectral laser source with a narrow spectral linewidth. For example, the *FSR_LM_* of the multiple longitudinal modes is 20 MHz when the round-trip length of the laser cavity is 10.4 m, as shown in Figure 1a. The etalon filter with a 4.16 mm cavity separation has fixed transmission peaks at 50 GHz intervals of *FSR_EF_* and 6.9 GHz of full width at half maximum (FWHM_EF_), as shown in Figure 1b. It is evident that the matching spectra among the multiple peak positions can be amplified repeatedly, while the spectra without transmission overlapping are relatively suppressed. Figure 1c shows that the transmission spectrum of the conventional WSL is mainly influenced by the spectral summation of the longitudinal-mode positions and the shifted transmission wavelength of the AOTF. If we sweep the laser output of the conventional WSL, the optical intensity will be continuously maintained throughout the examined time scale because there are many multiple longitudinal modes in a single peak of laser output transmission. 

In comparison, the comb-like laser output with a narrow spectral linewidth is explained in Figure 1d, which shows that the proposed WCSL is instead determined by the spectral cascading positions of the static longitudinal mode, the static etalon filter, and the dynamic AOTF. The transmission spectra swept by the AOTF for both WSL and WCSL, obtained after a time interval Δt, which is related to the *FSR_EF_* of the etalon filter, are also shown with dashed lines in Figure 1c,d, respectively. Among the three peaks of Figure 1d, the highest one results from the full overlapping between the peak spectrum of the AOTF and the etalon filter, while the two smaller peaks correspond to the partial overlapping between the side-slope of the AOTF and the etalon filter. This means that the optical intensity will also have a comb-like pulse shape along the time scale when sweeping the laser output of the proposed WCSL.

Assuming that the spectral linewidth of the conventional WSL in Figure 1c is narrower than the *FSR_EF_* of the etalon filter, only one strong spectrum will be generated in Figure 1d. This means that when multiple peaks exist in the WCSL output by inserting the etalon filter, the spectral linewidth of the WCSL becomes narrower than that of the conventional WSL. As a result, extended displacement measurements will be possible in SS-OCT using the WCSL with the AOTF when the coherence length of the WCSL can be enhanced by the etalon filter compared to that of the conventional WSL with the AOTF. To avoid the comb-like output spectrum, a narrower spectral width of AOTF and a wider *FSR_EF_* of the etalon filter can be applied to expect the single-mode operation of the laser output. However, a comb-like pulse shape will still be maintained along the time for the wavelength-stepped laser output [8,9].

## 3. Experimental Setups of WCSL Source and Interferometric Displacement Measurement

### 3.1. Experimental Setup of WCSL Source

**Figure 2:**
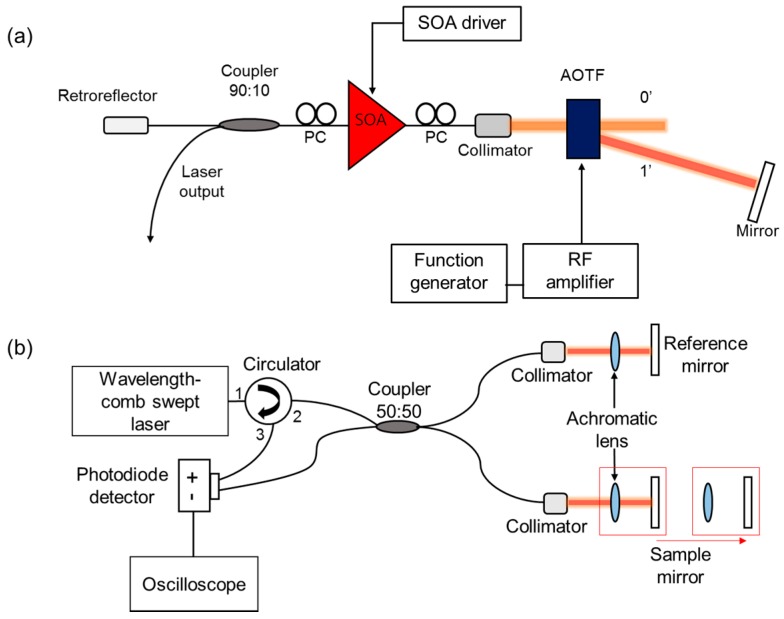
(**a**) Experimental setup of the WCSL with the AOTF; SOA: semiconductor optical amplifier, PC: polarization controller, AOTF: acousto-optic tunable filter; (**b**) Experimental setup for the measurement of the PSF.

Figure 2a shows the experimental setup of the WCSL with the AOTF which includes an external tunable laser cavity structure with a linear cavity. The cavity consists of two optical media: a single-mode fiber part and a free-space part. The single-mode fiber part has a fiber-coupled semiconductor optical amplifier (SOA) as the gain medium, two polarization controllers (PCs) for controlling the polarization state, a 90:10 output coupler to induce the laser output with a 10% port, and a fiber-coupled retro-reflector for back-reflection. The free-space part has a fiber-coupled collimator that propagates the light from the single-mode fiber to free space, an AOTF for the selection of the output wavelength by the RF electric signal, an etalon filter to induce fixed transmission peaks with 50 GHz of *FSR_EF_* and 6.9 GHz of FWHM_EF_, and a mirror for the back-reflection of the filtered spectrum by both the AOTF and the etalon filter. When the etalon filter is removed, the laser operates as a conventional WSL without a comb-like spectral output. Because the AOTF is driven by a function generator and an RF amplifier, there are no mechanical moving parts used during the shifting of the output wavelength of the WCSL. Compared to the uni-directional ring-type cavity, the linear-cavity-type configuration has some merits, such as higher output power, improved signal-to-noise ratio (SNR), and broader sweeping bandwidth [19]. 

### 3.2. Experimental Setup for Interferometric Displacement Measurement

Figure 2b shows the interferometric experimental setup for the measurement of the PSF. The interferometer is based on a Michelson interferometer and consists of a circulator, a 50:50 coupler, a reference arm, and a sample arm with a motorized translation stage. Both arms include a collimator, an achromatic lens, and a mirror to tune the path length difference in the free-space range. The path length of the reference arm is fixed, whereas the path length of the sample arm is changed by moving the achromatic lens and mirror on the translation stage with a linear motor. The period of the interferometric spectrum is inversely proportional to the optical path length difference between the reference and sample arms. The optical interferometric signal is converted to an electric signal using a balanced photodetector, and the signal is recorded with an oscilloscope and transformed to the PSF for the measurement of the path length difference. 

## 4. Laser Performance and Interferometric Displacement Measurement Results

### 4.1. Comparison of Output Performance between Conventional WSL and Proposed WCSL 

**Figure 3:**
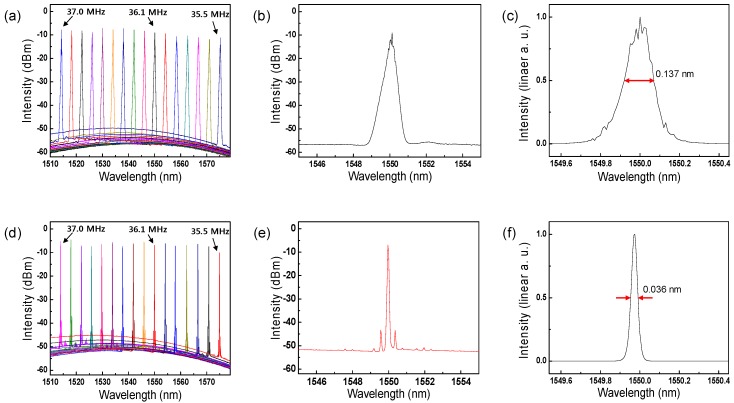
(**a**–**c**) Conventional WSL with AOTF; (**d**–**f**) proposed WCSL with AOTF. (**a**,**d**) Static output spectra for various RF signal; (**b**,**e**) dB-scale spectra at 1550 nm for shape monitoring; (**c**,**f**) linear-scale spectra at 1550 nm for linewidth measurement.

**Figure 4:**
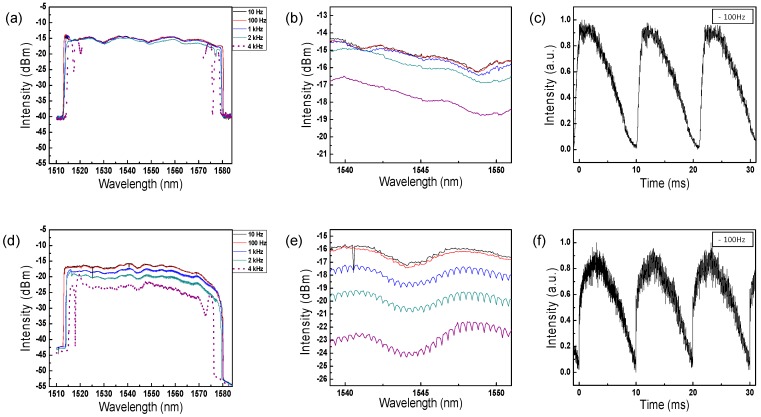
(**a**–**c**) Conventional WSL with AOTF; (**d**–**f**) proposed WCSL with AOTF; (**a**,**d**) Dynamic sweeping output spectra with a repetition rate of 10 Hz, 100 Hz, 1 kHz, 2 kHz and 4 kHz; (**b**,**e**) magnified spectra from 1539 to 1551 nm. (**c**,**f**) Time-trace measurement of the sweeping output at a repetition rate of 100 Hz.

Figure 3 displays the static output measurements of the two built lasers, WSL and WCSL, and shows the spectral difference obtained by the insertion of the etalon filter in the cavity, as shown in Figure 2a. Figure 3a–c presents the measurement results when the etalon filter is not inserted in the cavity. Because the frequency of the RF signal applied to the AOTF is inversely proportional to the output wavelength, Figure 4a shows that the center of the output wavelength is continuously swept from 1579.4 to 1514.36 nm when an RF signal from 35.4 to 37.0 MHz, respectively, is applied. The bandwidth of the overall tuning range is approximately 65 nm and all SNRs are higher than 50 dB. Figure 3b is a dB-scale spectral shape of the laser output at a center wavelength of 1550 nm, and Figure 3c is the corresponding linear-scale representation that displays the spectral linewidth of 0.137 nm more clearly. It must be mentioned that the measured spectral linewidth of 0.137 nm is a reliable result because the specifications of the OSA used in this experiment (MS9740A, Anritsu, Kanagawa Prefecture, Japan) stated a spectral resolution of 0.03 nm. 

Figure 3d–f shows the results of the WCSL with the etalon filter of a 50 GHz *FSR_EF_* operating as a comb filter. Figure 3d shows that when an RF signal from 35.5 to 37.0 MHz is applied continuously, the output wavelength is generated discretely from 1574.88 to 1513.92 nm with a comb-like pulse shape. In Figure 3a, it is observed that the bandwidth measurement of 61 nm becomes narrower and the SNR of 45 dB decreases by the insertion loss and reduced gain due to the additional etalon filter. The output power of the lasers is 3.8 mW for the WSL and 2.7 mW for the WCSL under the same conditions, with a driving current of 300 mA on the SOA. From Figure 3e, three comb-like peaks are clearly observed with a dB-scale spectral shape. This results from the combination of the longitudinal mode, etalon filter, and output transmission of the conventional WSL with the AOTF. Figure 3f shows the measurement result of the significantly reduced linewidth of 0.036 nm, which is almost similar to the spectral resolution limit of the OSA [10,16].

**Figure 5:**
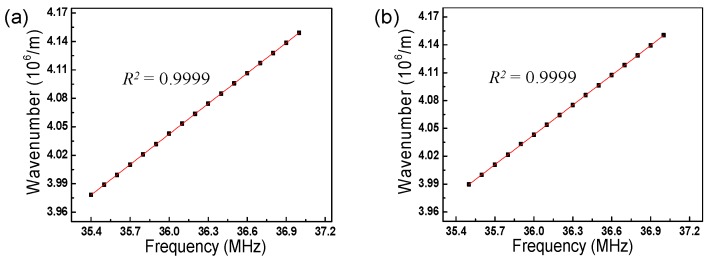
Linear relationship between applied RF signal and output wavenumber: (**a**) conventional WSL with AOTF; (**b**) proposed WCSL with AOTF.

The sweeping range of the dynamic output of the two lasers was also measured in the peak-hold mode of the OSA to observe whether these lasers can sweep the entire tunable wavelength region of the static laser output. Figure 4a–f shows the dynamic sweeping output spectra with a repetition rate of 10 Hz, 100 Hz, 1 kHz, 2 kHz and 4 kHz for the conventional WSL with an AOTF and the proposed WCSL with an AOTF. As the repetition rate increases, the swept wavelength range narrows, as seen in Figure 4a,d. With our cavity configuration, unstable sweeping is monitored when the repetition rate increases above 4 kHz. Because the maximum repetition rate for stable sweeping is reduced by the total round-trip cavity length, the stable sweeping speed can be easily increased by reducing the cavity length of the laser [20]. By closely comparing the spectra between 1539 and 1551 nm, Figure 5e clearly shows a comb-like spectrum generated by the *FSR_EF_* of the etalon filter, unlike Figure 5b.

Figure 4c,f shows the time trace of the sweeping output when the two types of lasers, WSL and WCSL, are driven by a triangular waveform with a repetition rate of 100 Hz. Because a comb-like spectral output is generated in the WCSL by the combination of the AOTF and the etalon filter, as shown in Figure 1d, we can clearly observe a repeated comb-like intensity output in each repeated cycle in Figure 4e [5]. The time-period of the cycles is determined by the repetition rate of the AOTF, while that of the comb-like pulse in each cycle is determined by the *FSR_EF_* of the etalon filter.

Because the proposed WCSL, which is based on an AOTF, has no mechanical moving parts for the selection of the wavelength and is solely driven by an electro-optic signal, it has some advantages in comparison to other types of WCSLs using an FFP-TF, such as high environmental stability, wavenumber linearity, and adaptability to various types of driving functions [5,6,7,8]. Figure 5 shows the linear relationships between the applied RF signal and the output wavenumber [14] for both WSL and WCSL, which have a high wavenumber linearity of *R*^2^ = 0.9999. When the WCSL based on the AOTF has a high wavenumber linearity, it does not need an additional process for the *k*-linear spacing of the interferometric signal to ensure equally spaced wavelength combs in the wavenumber domain during mass signal processing for imaging and sensing.

### 4.2. Comparison between Interferometric Displacement Measurements with Conventional WSL and Proposed WCSL 

**Figure 6:**
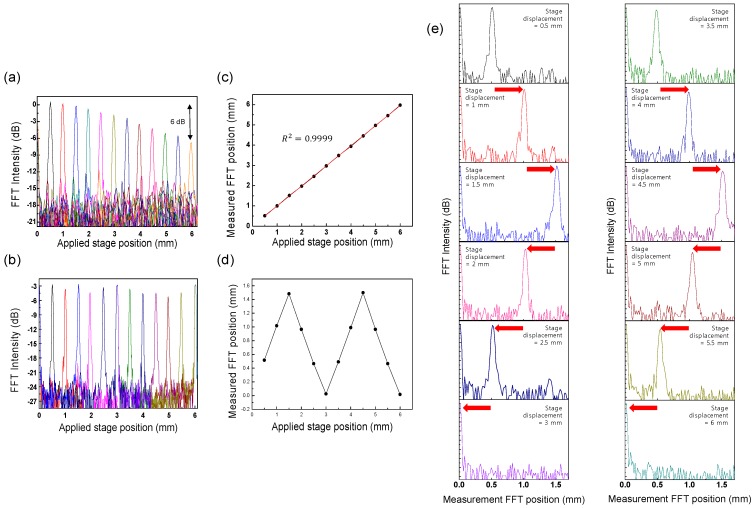
Results obtained when the stage displacement changes from 0.5 to 6 mm. (**a**) Trace of the FFT intensity of the conventional WSL as a function of the applied stage position; (**b**) Trace of the FFT intensity of the proposed WCSL for the applied stage position; (**c**) The relation between the applied stage position and measured FFT position of the conventional WSL; (**d**) The relation between the applied stage position and measured FFT position of the proposed WCSL; (**e**) Trace of the FFT intensity of the proposed WCSL for the measured FFT position.

The displacement measurement can be simply performed by measuring the fast Fourier transform (FFT) position of the PSF using the interferometric measurement setup of Figure 2. When changing the applied stage position, we can easily modify the optical path length difference between the reference arm and the sample arm. At every sweeping repetition of WCSL, the optical interferometric signal in the time domain is converted to the frequency domain by implementing the FFT process. As a result, the peak frequency of the FFT intensity is acquired as the measured FFT peak position of the mirror displacement of the translation stage in the sample arm. Figure 6a shows the PSF distribution of various FFT intensity measurements using the conventional WSL with the AOTF. As the applied stage position is increased from 0.5 to 6 mm, the peak position of the measured FFT position has the same value with the applied stage position as shown in Figure 6c. As the sensitivity roll-off of the FFT intensity is continuously measured, it is observed that the FFT intensity difference between the stage positions of 0.5 mm and 6 mm is 6 dB. It means that the coherence length of the conventional WSL can be considered to be double 5.5 mm, that is, approximately 11 mm [21].

Figure 6b shows the PSF measurement for each applied stage position of the proposed WCSL for longer displacement measurements. Compared to Figure 6a, the total FFT intensity is reduced to approximately 3 dB because of the reduced output power of the light source; however, the sensitivity roll-off is repeatedly decreased or increased until the applied stage position reaches 6 mm. Therefore, we expect that measurement at longer displacements is possible with minimal sensitivity loss of the optical fringe decay because of the narrower bandwidth of the wavelength-comb spectrum and the longer coherence length of the WCSL. 

However, it is also necessary to consider the sub-sampling effect of the wavelength-comb spectrum and the corresponding alias fringe signal in the extended displacement range. Because the *FSR_EF_* of the etalon filter in our WCSL was 50 GHz, the principal measurement range was limited to 0–1.5 mm and reflection from outside this principal range appeared aliased back to the principal range. This phenomenon is experimentally explained in detail in Figure 6e, where the trace of the FFT intensity is displayed along the measured FFT position, instead of the applied stage position of Figure 6b. As the applied stage position changes from 0 to 1.5 mm, the peak of the FFT intensity also moves along the measured FFT position axis as shown in Figure 6d. However, when the applied stage position exceeds 1.5 mm, the peak of FFT intensity returns to the decreased value of the measured FFT position axis until zero. Next, after increasing the stage position from 3 mm to 4.5 mm, the peak of the FFT intensity approaches the measured FFT position of 1.5 mm again, in starting from 0 mm. This means that the round trip of the PSF between 0 and 1.5 mm of the measured FFT position occurs for every 3 mm increase of the applied stage position. Because the stage positions of 0.5, 2.5, 3.5 and 5.5 mm have the same PSF peak in the measured FFT position at 0.5 mm, a de-aliasing algorithm can be used to distinguish the mirror PSF from the original PSF during the sub-sampling process [5,8,9]. 

**Figure 7:**
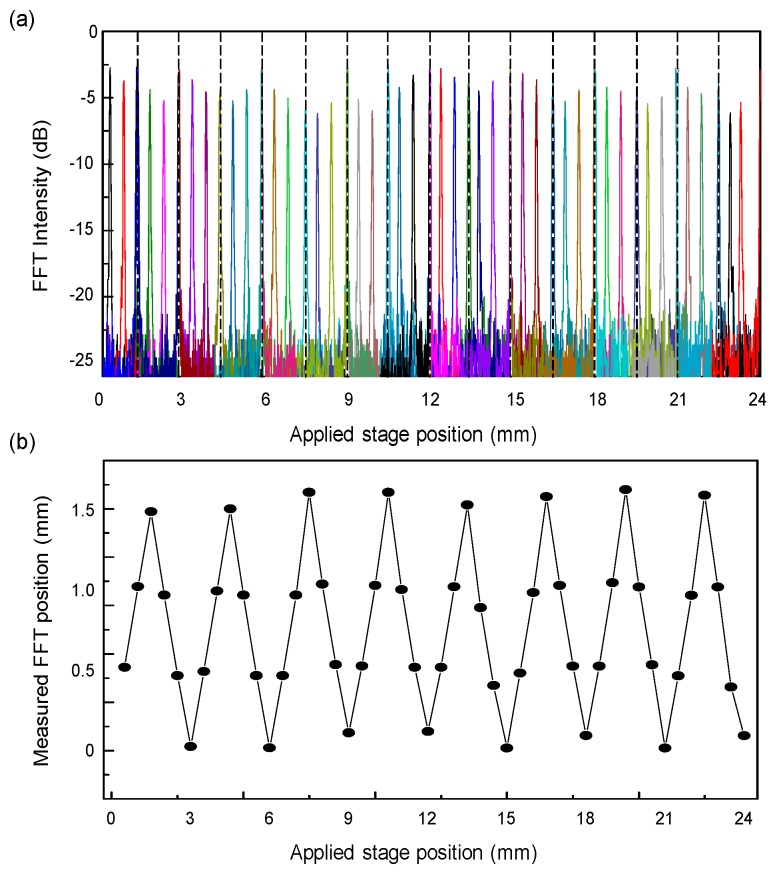
(**a**) Trace of FFT intensity of the proposed WCSL for applied stage positions from 0.5 to 23.5 mm; (**b**) Relationship between the applied stage position and the measured FFT position.

Figure 7a shows the trace of the FFT intensities of the WCSL for extended displacement measurements at stage positions from 0.5 to 24 mm. Because the maximum limitation of our translation stage displacement was 24 mm, it was difficult to measure above 24 mm; however, longer displacement measurements will be available as long as the path length difference reaches below the coherence length of the WCSL light. In our experimental setup using a different translation stage at a longer position, the optical interferometric signal can be clearly measured over 50 mm of displacement. However, it is not easy to get a continuously connected PSF distribution over 24 mm when the optical alignment between translation stages is not guaranteed. For the quantitative confirmation of the maximum range of the system, it is further necessary to measure the coherence length of the WCSL light with additional experiments with variable interferometer and signal analysis [22]. 

The relationship between the applied stage position and the measured FFT position is shown in Figure 7b. Because a single measured FFT position matches multiple target position values at a time, one can guarantee the distribution of absolute measurement only when the displacement change on the target surface is less than 1.5 mm. However, it also means that the maximum measurable range of the target position can be extended up to 24 mm and the loss of sensitivity is also reduced for the optical interference signal coming a from longer distance. For the estimation of the measurement resolution of the system, the peak width of the PSF is measured to ~48 μm in Figure 7a, which matches well with the theoretical expectation from the full 60 nm sweep of the WCSL. For the absolute measurement system to allow only one measurement value at a time, the aliasing effect should be solved by the unwrapping method [8,9]. In case the displacement change is non-discrete and unidirectional in the distance of the path length difference, the continuous monitoring of the FFT intensity movement in the forward or backward direction can only be useful to obtain the absolute applied stage position information without de-aliasing. It is expected that an extended displacement measurement can be implemented using a modification of the WCSL based on the AOTF and the etalon filter in the laser cavity. 

## 5. Conclusions

In this study, we proposed an acousto-optic wavelength-comb-swept laser for extended displacement measurements by implementing a comb-like spectral laser combining an AOTF and an etalon filter. Compared to a conventional WSL with a coherence length of 11 mm, the WCSL has a narrower spectral linewidth and enhanced coherence length and it can measure the limited displacement change at the longer displacements position above 24 mm. Because the wavelength sweeping is based on the acousto-optic selection and no mechanical moving parts exist in the WCSL, the WCSL exhibits high *k*-linearity (*R*^2^ = 0.9999) for a simplified data process in comparison with the swept laser with an FFP-TF. 

## References

[1] Soller B.J., Gifford D.K., Wolfe M.S., Froggatt M.E. (2005). High resolution optical frequency domain reflectometry for characterization of components and assemblies. Opt. Express.

[2] Yasuno Y., Madjarova V.D., Makita S., Akiba M., Morosawa A., Chong C., Sakai T., Chan K.-P., Itoh M., Yatagai T. (2005). Three-dimensional and high-speed swept-source optical coherence tomography for in vivo investigation of human anterior eye segments. Opt. Express.

[3] Huber R., Wojtkowski M., Taira K., Fujimoto F.G., Hsu K. (2005). Amplified, frequency swept lasers for frequency domain reflectometry and OCT imaging: Design and scaling principles. Opt. Express.

[4] Jung E.J., Park J.-S., Jung M.Y., Kim C.-S., Eom T.J., Yu B.-A., Gee S., Lee J., Kim M.K. (2008). Spectrally-sampled OCT for sensitivity improvement from limited optical power. Opt. Express.

[5] Tsai T.-H., Zhou C., Adler D., Fujimoto J. (2009). Frequency comb swept laser. Opt. Express.

[6] Tomasz B., Maciej W., Maciej S., Anna S., Robert H., Andrzej K. (2008). Improved spectral optical coherence tomography using optical frequency comb. Opt. Express.

[7] Zhang Y., Zhang Y., Zhao Q., Li C., Yang C., Feng Z., Deng H., Zhou E., Xu X., Wong K.K.Y., Yang Z. (2016). Ultra-narrow linewidth full C-band tunable single-frequency linear-polarization fiber laser. Opt. Express.

[8] Siddiqui M., Vakoc B.J. (2012). Optical-domain subsampling for data efficient depth ranging in Fourier-domain optical coherence tomography. Opt. Express.

[9] Tozburun S., Siddiqui M., Vakoc B.J. (2014). A rapid, dispersion-based wavelength-stepped and wavelength-swept laser for optical coherence tomography. Opt. Express.

[10] Masahiro U., Yuichi O., Seiji T., Takashi S., Yuzo S., Junya K., Kazunori N., Shogo Y. (2013). Improvement of coherence length in a 200 kHz swept light source with a KTa_1−*x*_Nb_*x*_O_3_ deflector using an etalon. Appl. Phys. Express.

[11] Eigenwillig C.M., Biedermann B.R., Palte G., Huber R. (2008). K-space linear Fourier domain mode locked laser and applications for optical coherence tomography. Opt. Express.

[12] Jeon M., Kim J., Jung U., Lee C., Jung U., Boppart S.A. (2011). Full-range k-domain linearization in spectral-domain optical coherence tomography. Appl. Opt..

[13] Hu Z., Rollins A.M. (2007). Fourier domain optical coherence tomography with a linear-in-wavenumber spectrometer. Opt. Lett..

[14] Han G.-H., Cho S.-W., Park N.S., Kim C.-S. (2016). Electro-optic swept source based on AOTF for wavenumber-linear interferometric sensing and imaging. Fibers.

[15] Wang Q., Zhang Y., Liu W.-H. (2006). Fabry-perot etalon filter. Proc. SPIE.

[16] Zhang D., Zhao J., Yang Q., Liu W., Fu Y., Li C., Luo M., Hu S., Hu O., Wang L. (2012). Compact MEMS external cavity tunable laser with ultra-narrow linewidth for coherent detection. Opt. Express.

[17] Merlier J.D., Mizutani K., Sudo S., Sato K., Kudo K. (2006). Wavelength channel accuracy of an external cavity wavelength tunable laser with intracavity wavelength reference etalon. J. Lightwave Technol..

[18] Sato K., Mizutani K., Sudo S., Tsuruoka K., Naniwae K., Kudo K. (2007). Wideband external cavity wavelength-tunable laser utilizing a liquid-crystal-based mirror and an intracavity etalon. J. Lightwave Technol..

[19] Dong X., Ngo N.Q., Shum P., Tam H.-Y., Dong X. (2003). Linear cavity erbium-doped fiber laser with over 100 nm tuning range. Opt. Express.

[20] Huber R., Wojtkowski M., Fujimoto J.G. (2006). Fourier domain mode locking (FDML): A new laser operating regime and applications for optical coherence tomography. Opt. Express.

[21] Yamashita S., Takubo Y. (2013). Wide and fast wavelength-swept fiber lasers based on dispersion tuning and their application to optical coherence tomography. Photonic Sens..

[22] Salvade Y., Przygodda F., Rohner M., Polster A., Meyer Y., Monnerat S., Gloriod O., Llera M., Matthey R., Francisco J. D. (2016). Interferometric measurements beyond the coherence length of the laser source. Opt. Express.

